# Stretching the Limits: A Rare Case Report of Perimesencephalic Subarachnoid Hemorrhage During Yoga

**DOI:** 10.5811/cpcem.53231

**Published:** 2026-06-27

**Authors:** Andrew Vierra, Mrinal Sinha, Leena Jamal, Nadir Khan, Taylor Liebert, Abdullah Bokhari

**Affiliations:** * McLaren Oakland Hospital, Department of Emergency Medicine, Pontiac, Michigan; † Michigan State University College of Osteopathic Medicine, East Lansing, Michigan

**Keywords:** subarachnoid hemorrhage, perimesencephalic hemorrhage, yoga, Valsalva maneuver, case report

## Abstract

**Introduction:**

Nontraumatic subarachnoid hemorrhage (SAH) is a life-threatening neurological emergency, accounting for 5–10% of strokes. While most cases result from aneurysm rupture, perimesencephalic nonaneurysmal SAH is a distinct subtype characterized by blood localized to the midbrain cisterns and a venous pathophysiology. Perimesencephalic nonaneurysmal SAH is often associated with activities increasing intracranial venous pressure. Reports linking it to low-impact exercise such as yoga are rare.

**Case Report:**

A 45-year-old female with no significant medical history presented with acute onset of severe headache and subjective unilateral hearing loss. Symptoms began immediately after a sudden, startled hyperextension movement during a yoga session. Noncontrast computed tomography of the brain revealed hemorrhage localized to the perimesencephalic and basal cisterns (modified Fisher Grade 4). Magnetic resonance imaging confirmed hemorrhage extension into the fourth ventricle and communicating hydrocephalus. Comprehensive workup, including echocardiography and serial digital subtraction angiography, ruled out aneurysm, arteriovenous malformation, or cardiac source.

**Conclusion:**

The patient was diagnosed with perimesencephalic nonaneurysmal subarachnoid hemorrhage, likely precipitated by an abrupt Valsalva-like maneuver during yoga. She was managed conservatively with strict blood pressure control, chemical thromboprophylaxis, and vasospasm prophylaxis, achieving a full recovery. This case highlights that neurovascular events can occur during low-impact activities in susceptible individuals. Recognition of a distinct radiological pattern of perimesencephalic nonaneurysmal—confirmed by negative serial angiography—is vital, as it carries a significantly more favorable prognosis than aneurysmal SAH, allowing for conservative management and reassurance.

## INTRODUCTION

Subarachnoid hemorrhage (SAH) represents a critical cerebrovascular event associated with significant morbidity and mortality. Approximately 85% of nontraumatic SAH cases are caused by the rupture of a saccular aneurysm, presenting classically as a “thunderclap” headache. However, perimesencephalic nonaneurysmal SAH represents a unique subset, accounting for roughly 10% of all SAH cases and up to two-thirds of those with negative initial angiography.^1^,^2^ Unlike aneurysmal SAH, which is arterial in origin, the pathophysiology of perimesencephalic nonaneurysmal SAH is believed to involve a “venous component.” Current literature suggests that rupture of small perimesencephalic veins or capillary beds occurs due to sudden spikes in intracranial venous pressure. These pressure surges are often triggered by Valsalva maneuvers, such as coughing, heavy lifting, or straining. 3,4

While SAH is frequently associated with high-intensity physical exertion, its occurrence during low-impact activities such as yoga is rarely documented in the literature. This report describes a case of angiogram-negative perimesencephalic nonaneurysmal SAH precipitated by an abrupt movement during yoga. Here we highlight the importance of distinguishing this condition from aneurysmal causes and discuss the osteopathic and clinical implications of this rare presentation.

## CASE REPORT

A 45-year-old female presented to the emergency department via emergency medical services with a chief complaint of acute, severe headache. The patient reported she had been performing yoga when she executed a sudden, reactive jump and back-extension movement in response to an unexpected environmental stimulus. Immediately following this maneuver, she experienced a sharp occipital headache, describing the pain as “throbbing” and rated 8/10 in severity. She noted associated lightheadedness, nausea, and the acute onset of subjective right-sided hearing loss. She denied direct head trauma or loss of consciousness. The patient’s past medical history was unremarkable. On arrival, her vital signs were within normal limits: blood pressure, 123/80 millimeters of mercury (mmHg); heart rate, 86 beats per minute; respiratory rate, 17 breaths per minute; and oxygen saturation, 99 % on room air. The patient was afebrile, temperature 36.9 °Celsius.

Physical examination revealed limited range of motion of the cervical spine secondary to pain and tenderness in the right paraspinal musculature, most likely nuchal rigidity. Neurological examination yielded a National Institutes of Health Stroke Scale score of 0. The patient was alert and oriented to person, place and time with no focal motor or sensory deficits. Bedside auditory testing with finger rub revealed gross deficits in right-sided hearing, which were transient.

**Image 1:**
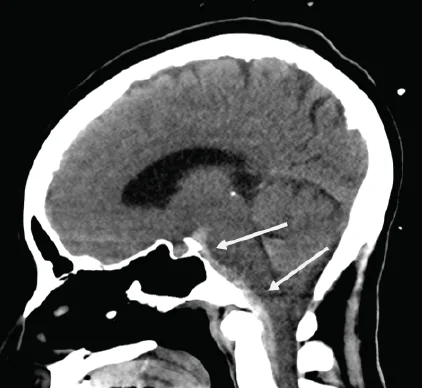
Computed tomography of the head (sagittal view) demonstrating hyperattenuation within the prepontine cistern, which extends inferiorly to the upper cervical spine relating to subarachnoid hemorrhage (arrows) in a patient diagnosed with perimesencephalic nonaneurysmal subarachnoid hemorrhage.

**Image 2:**
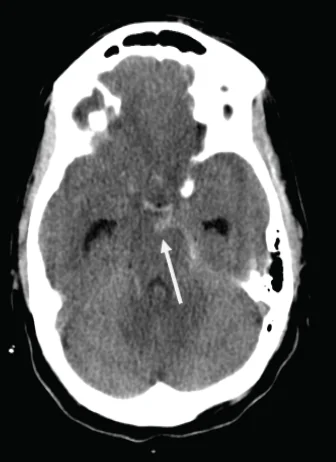
Computed tomography of the head (axial view) showing hemorrhage extension into the perimesencephalic cisterns within the prepontine cistern, slightly asymmetric to the left (arrow).

**Image 3:**
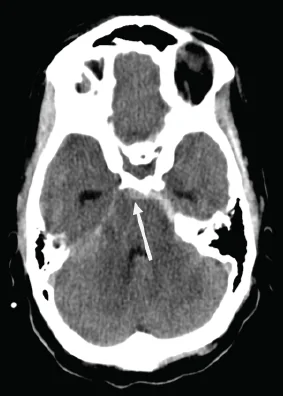
Computed tomography of the head (axial view) at the level of the foramen magnum within the prepontine cistern showing hemorrhage into the spinal canal (arrow) in a patient diagnosed with perimesencephalic nonaneurysmal subarachnoid hemorrhage.

**Image 4:**
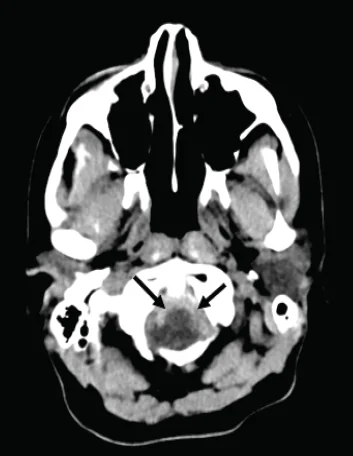
Computed tomography of the head (axial view) at the level of the upper cervical spine showing hemorrhage into the spinal canal (arrows).

An emergent noncontrast computed tomography of the brain demonstrated SAH localized within the suprasellar, prepontine, and interpeduncular cisterns (Images 1 and 2). The hemorrhage extended into the cistern of Liliequist, the fourth ventricle, and tracked into the upper cervical spinal canal (Images 3 and 4). There was effacement of the bifrontal sulci and mild ventricular prominence. The imaging findings were classified as modified Fisher Grade 4. Computed tomography angiography of the head and neck revealed no evidence of high-grade arterial stenosis, occlusion, intracranial aneurysm, or arteriovenous malformation.

*CPC-EM Capsule*

What do we already know about this clinical entity?

*Perimesencephalic nonaneurysmal subarachnoid hemorrhage (SAH) is a non-aneurysmal subtype of SAH with a venous origin and more favorable prognosis than aneurysmal SAH.*

What makes this presentation of disease reportable?

*We document a rare case triggered by a sudden Valsalva maneuver during yoga, with atypical symptom of acute hearing loss.*

What is the major learning point?

*It arises from venous rupture, often occult on angiography. Diagnosis relies on the distinct prepontine pattern on CT despite negative vessel imaging.*

How might this improve emergency medicine practice?

*Identifying the distinct pattern of perimesencephalic nonaneurysmal SAH allows for conservative management and avoids unnecessary interventions.*

The patient was transferred to a tertiary care center for neurocritical care and neurosurgery consultation. She was admitted to the neuro-intensive care unit with a Hunt and Hess Grade 1. Magnetic resonance imaging of the brain confirmed SAH along the prepontine cistern and foramen magnum involving the fourth ventricle. Fluid-attenuated inversion recovery sequencing demonstrated linear increased signal intensity in the bilateral frontal and parietal-occipital regions, suggesting additional foci of hemorrhage. Dilation of the temporal horns suggested an element of communicating hydrocephalus.

The patient underwent an initial digital subtraction angiography, which was negative for aneurysm or arteriovenous malformation. A repeat inpatient digital subtraction angiography performed was also negative. A transthoracic echocardiogram revealed a normal ejection fraction (60–65 %) and a negative bubble study, ruling out a patent foramen ovale or cardiac shunt. Transcranial Doppler was performed daily and remained negative for vasospasm.

Management focused on strict hemodynamic control. Systolic blood pressure was maintained at < 140 mm Hg using a nicardipine infusion (titrated by 2.5 mg/hour every 15 minutes), which was weaned as tolerated. Nimodipine 60 mg every four hours was initiated for a 21-day course for vasospasm prophylaxis. Levetiracetam 500 mg twice daily was administered for seven days for seizure prophylaxis. Mannitol 25 grams every six hours was administered; although the patient remained alert, this was used for symptomatic headache relief and to optimize intracranial pressure, given the mild ventricular prominence and bifrontal sulcal effacement seen on imaging. Fluid status was maintained net positive. Bilateral sequential compression devices were applied initially. Once deemed safe by neurosurgery (approximately 48 hours post-admission), subcutaneous heparin, 5,000 units every eight hours, was initiated. An admission lipid panel revealed a low-density lipoprotein (LDL) of 89.4 milligrams per deciliter (mg/dL) (reference range: 0–99 mg/dL). Atorvastatin 40 mg daily was initiated with a target LDL < 70 mg/dL.

The patient remained neurologically stable throughout her admission. Her headache improved with a regimen of acetaminophen, butalbital/acetaminophen/caffeine, and avoidance of stimulation. She did not require external ventricular drainage. She was discharged home in stable condition. At her outpatient follow-up, she underwent a third digital subtraction angiography, which remained negative, confirming the diagnosis of cryptogenic/nonaneurysmal SAH. She reported no recurrence of “thunderclap” headaches, complete resolution of hearing symptoms, and had successfully returned to work.

## DISCUSSION

This case illustrates a rare presentation of spontaneous perimesencephalic nonaneurysmal SAH triggered by a sudden hyperextension movement during yoga. The clinical and radiographic findings align with the diagnostic criteria: hemorrhage centered anterior to the midbrain, absence of aneurysm on angiography, and a benign clinical course (Hunt and Hess Grade 1).

Spontaneous perimesencephalic nonaneurysmal SAH with venous components triggered by a sudden, non-traumatic movement during a low-impact activity is rare. The pathophysiology is distinct from aneurysmal rupture. The “venous hypothesis” postulates that the hemorrhage results from the rupture of superficial perimesencephalic veins or the basal vein of Rosenthal. In this case, the sudden back extension and likely simultaneous breath-holding (Valsalva maneuver) caused a transient spike in intrathoracic pressure. This pressure is transmitted retrograde through the jugular venous system to the intracranial veins, which lack valves, potentially causing a thin-walled vein to rupture. While exercise-induced SAH is documented, yoga is generally considered a low-risk activity. This case suggests that specific rapid movements can generate sufficient physiological stress to trigger perimesencephalic nonaneurysmal SAH in susceptible individuals.^3^,^4^

A notable strength of this case is the extensive workup, which excluded other etiologies. The patient underwent three separate digital subtraction angiograms (the gold standard) over a distinct time course, all of which were negative. Furthermore, magnetic resonance imaging ruled out occult cavernomas, and the negative bubble study on echocardiogram ruled out paradoxical embolism. This rigorous exclusion supports the diagnosis of a venous/cryptogenic etiology.

The presentation with unilateral hearing loss is atypical yet not an uncommon feature of SAH. This can be attributed to several mechanisms: direct irritation of the vestibulocochlear nerve as it traverses the blood-filled cisterns, or a central auditory processing disorder caused by the toxic effects of blood products (hemosiderin) on the auditory cortex.^5^ The complete resolution of her hearing deficit suggests the cause was likely transient nerve irritation rather than permanent central damage.

Perimesencephalic non-aneurysmal SAH accounts for approximately 10% of all SAH cases and two-thirds of angiogram-negative SAH cases.^3^ Its distinction from aneurysmal SAH is critical due to its vastly different prognosis. Patients with perimesencephalic nonaneurysmal SAH have a much lower risk of re-bleeding, symptomatic vasospasm, and long-term disability, and the mortality rate is near zero.^3^,^6^ This contrasts sharply with the potential for devastating outcomes after an aneurysmal rupture.

## CONCLUSION

Emergency physicians should maintain a high index of suspicion for subarachnoid hemorrhage in patients presenting with sudden, severe, “thunderclap” headache, even following low-impact activities like yoga. This case of perimesencephalic nonaneurysmal SAH occurring during yoga demonstrates a rare trigger with an atypical presentation. It reinforces the favorable prognosis of this condition when serial angiography is negative, supporting a conservative management strategy focusing on blood pressure control, chemical thromboprophylaxis, and vasospasm prophylaxis. Recognizing the characteristic imaging pattern of perimesencephalic nonaneurysmal SAH is essential for accurate diagnosis and prognosis, guiding management toward a conservative approach and providing reassurance regarding the typically favorable long-term outcomes.
